# Analysis of Survival Rates of Patients Diagnosed With Incipient Esophagic Cancer

**DOI:** 10.4021/wjon2010.02.193w

**Published:** 2010-02-01

**Authors:** Edmundo Carvalho Mauad, Maria do Rosario Dias de Oliveira Latorre, Thiago Buosi Silva, Ricardo Mauad Daher, Vinicius de Lima Vazquez, Antonio Talvane Torres Oliveira, Adhemar Longatto Filho

**Affiliations:** a Barretos Cancer Hospital, Department of Cancer Screening, Sao Paulo, Brazil; b Laboratory of Medical Investigation (LIM) 14 of Department of Pathology of Medical School of Sao Paulo University, Sao Paulo, Brazil; c Life and Health Sciences Research Institute (ICVS), School of Health Sciences, University of Minho, Braga, Portugal

**Keywords:** Cancer survival rate, Insipient esophageal cancer, Esophagectomy, Cancer treatment

## Abstract

**Background:**

Esophagic cancer incidence is extremely variable worldwide. Also, the global survival rate has not oscillated significantly since last decade. Most of the worse prognoses are found among patients with advanced stages. Despite that, around 10% of cases occur in patients with initial stage, which strongly associate these patients with unfavorable prognosis. We sought to analyze the impact of time free of disease and global survival rates of patients with initial stage of esophagic cancer.

**Methods:**

We studied 18 patients with initial stage of esophagic cancer (stage 0 and I), examined and treated at Hospital de Cancer de Barretos between 1990 and 2005.

**Results:**

The vast majority of patients were male (83.3%) with age up to 49 yarest old (77.8%), squamous cell carcinoma (SCC) (88.9%) and stage I (83.3%). Most of them were smoker (60.0%) and etilist (62.5%). There were 38.9% of the patients with comorbities like dysphagia and epigastralgia correlated to other pathological conditions. We found free disease rates of 100% and 82.5%, respectively for 12 and 36 months. The significant prognostic evidence was the age, epigastralgia symptoms and chemotherapy. From 18 patients, 6 passed away during the period of 36 months follow up due to cancer consequences. The probabilities of global survival were 76.7% and 64.4% after 12 and 36 months, respectively, and none of the analyzed variables influenced in theses rates.

**Conclusions:**

Our data ratifies those from previous reported. The global survival rates were worse than reported by literature, maybe in consequence of the poor clinical condition of many patients which limited the option for more aggressive therapy.

## Introduction

Esophagic cancer incidence is extremely variable worldwide. In Europe, this type of cancer is uncommon, and the global survival rate has not oscillated significantly since last decade. The survival rates after five years fluctuate around 3% (for men in Slovenia or Italy) and 19% (for women in Finland) [1]. New 16,470 esophagic cancer cases are estimated in United States for 2008 and the number of deaths around to 14,200 [2]. Brazil usually have inferior rates of esophagic cancer, but in the Center-south region the incidence is 15 new cases for 100,000 men, and four new cases for 100,000 women [3].

Esophagic carcinomas are commonly detected in advanced stage when more than 75% of esophagic space is blocked by the tumor. Consequently, dysphagia and pronounced weight losing are the principal symptoms observed in these patients [4, 5]. The concomitant consumptions of alcohol and tobacco are significantly associated to the esophagic carcinogenesis [6].

Endoscopy screening is indicating to identify incipient lesions of esophagic tumor favoring more efficient treatment and best prognostic outcome. Japan studies demonstrated that screening endoscopic program detected almost 30% of esophagic carcinoma staged I and II in asymptomatic individuals [7, 8]. Until 10 years ago, only 10% of all esophagic cancer was clinical stages (CS) 0 or 1. But this percentage has been increasing in the recent years. Important data emerged from implement screening programs to detect incipient forms of esophagic cancer. In United States, the global survival rates in five years increased from 6% in 1975 to 11% and 18% in 1984 and 2003, respectively. Data from EUROCARE have showed that survival rates in Europe is 33% after one year and 10% in 5 years with significant variation among different countries, additionally, it was demonstrated that there was no significant modification in the survival rates in two different periods: 1978 to 1980 and 1987 to1989 [9].

Importantly, patients with esophagic cancer detected in initial stages show survive rates of 88% after three years in German patients, 45% after 12 months in Iran, 67% in five years in Japan and 78.3% in Korea. Considering all stages of esophageal cancer from Singapore, no significant variations have been observed in mortality rates since the 70’s [10].

Most of the recurrence events are associated to the site of lesion development, type of the surgery used to remove the tumor, and multifocal lesions [11, 12]. Histological type of the tumor is also an important prognostic parameter [13]. Despite the importance of this tumor, Latin American data regarding outcome is not available. Most of the strategies to reduce mortality consequent to the esophageal cancer could be cost-effectively improved if endoscopic investigation in high risk population was rationally considered. A number of initial lesions could be identified and the therapeutic procedures are more efficient.

The objective of this study was to evaluate associated factors related with esophageal incipient cancer examined at Barretos Cancer Hospital (HCB) - Pio XII Foundation, between 1990 and 2005.

## Patients and Methods

The files of 1,853 esophageal cancer patients examined and treated in Barretos Cancer Hospital between 1990 and 2005 were revised. From these cases, 18 (1.0%) were categorized as Stage 0 and I (TNM classification). Histopathological classification of these tumors considered initial esophageal cancer cases of in situ carcinoma and those cases with mucosal and submucosal, but without lymph node invasion.

Surgical procedures comprised transhiatal and transthoracic esophagectomy, followed by peritumoral lymphadenectomy, and radiotherapy and or chemotherapy if necessary.

The following variables were analyzed, (1) socio-demographic: gender, ethnicity, age, schooling level, alcoholism and smoking status; (2) clinical: symptomatology, time relapsed since the first symptoms, Body Mass Index (BMI) and comorbidity; (3) pathological: second primary tumor, histological type, lesion size, and stage; (4) therapeutics: surgery, radiotherapy, chemotherapy; (5) follow up: dates of recurrence, death and last clinical visit.

### Statistical evaluation

Clinical and pathological data were stored and analyzed using the SPSS statistical software (version 16.0, SPSS Inc, Chicago, IL, USA). Free disease interval and global survival rates were calculated after intervals of 12 and 36 months using Kaplan-Meier and log rank test curves for data comparison.

## Results

Table 1 depicted the socio-demographic characteristics of the patients. The vast majority of the patients were men (15 cases, 83.3%) with age up to 49 years old (14 cases, 77.8%), had stage I (15 cases, 81.8%) and squamous cell carcinoma (16 cases, 88.9%). The majority of patients were alcoholic beverage consuming (10 cases, 62.5%) and smoker (9 cases, 60.0%), and 35.3% (6 cases) showed overweight. The symptoms more frequently reported were: dysphagia (12 cases, 66.7%), and weight loss (7 cases, 38.9%).

**Table 1:** Number and Percentage of Patients According to Socio-demographic and Clinical Characteristics, and Lifestyle

Variable	Category	n	%
Gender	Male	15	83.3
	Female	3	16.7
Age	≤ 49 years old	4	22.2
	≥ 50 years old	14	77.8
State of origin	Minas Gerais	5	27.8
	Mato Grosso do Sul	1	5.6
	Sao Paulo	12	66.7
Schooling	Non	5	27.8
	Basic incomplete	11	61.1
	Basic complete	1	5.6
	High school	1	5.6
Tabagism	Never/ Former smoker	9	60.0
	Current smoker	6	40.0
Etilism	Never	6	37.5
	Ever	10	62.5
Body Mass Index (BMI)	≤ 18.5	5	29.4
	18.5 – 25.9	6	35.3
	≥ 26.0	6	35.3
Comorbidity	Absent	11	61.1
	Present	7	38.9
Second primary tumor	Yes	5	27.8
	No	13	72.2
Histological type	Squamous cell carcinoma	16	88.9
	Adenocarcinoma	2	11.1
Stage (TNM)	0	3	16.7
	1	15	83.3
Therapeutics	Radiotherapy	4	22.2
	Surgery and radiotherapy	1	5.6
	Surgery and chemotherapy	1	5.6
	Radiotherapy and chemotherapy	1	5.6
	Transthoracic esophagectomy	4	22.2
	Transhiatal esophagectomy	6	33.3
	Esophageal gastroplasty	1	5.6
Total		18	100

Comorbidity was reported in 38.9% of the patients, and the principal diseases were Barrett's esophagus (2 cases, 11.1%), caustic soda ingestion (one case, 5.6%) and gastric ulcer (8 cases, 44.4%). Treatment varied depending on the characteristic of each patient and tumor. The most frequent options were: transhiatal esophagectomy (6 cases, 33.3%), transthoracic esophagectomy (4 cases, 22.2%), and radiotherapy (4 cases, 22.2%). Two patients (11.1%) were also treated with chemotherapy (one of them also treated by surgery and three by radiotherapy).

Anatomical location showed 1 (5.6%) case in superior esophageal area (20 to 25 cm from superior dental arcade, SDA), 8 (44.4%) cases in mid esophagus (25 to 30 cm from SDA) and 9 (50.0%) in inferior esophagus (30 to 40 cm from SDA).

Time of follow up ranged from one to 71 months. There were four cases of recurrence (23.5%), and free disease interval of 100% and 82.5%, for 12 and 36 months, respectively (Table 2). The significant prognostic factors were age (p = 0.009), epigastralgia (p = 0.038) and chemotherapy (p < 0.001).

**Table 2:** Free Disease Interval Rates Probabilities in Patients With Insipient Esophageal Cancer (Clinical Stage 0 and I - TNM) From Barretos Cancer Hospital, Diagnosed From 1996 To 2005

Variable	Category	n	Free disease rates probabilities (%)		p (Log-Rank)
			12 months	36 months	
Gender	Male	15	100.0	78.8	0.502
	Female	3	-	-	
Age	≤ 49 years old	4	100.0	33.3	0.009
	≥ 50 years old	14	-	-	
Schooling	Never	5	-	-	0.468
	Basic incomplete	11	100.0	87.5	
	Basic complete/ High school	2	100.0	50.0	
Tabagism	Never and/or former smoker	6	100.0	80.0	0.744
	Current smoker	9	100.0	75.0	
Etilism	Never	6	-	-	0.371
	Ever	10	100.0	80.0	
Weight loss	Yes	7	-	-	0.445
	No	11	100.0	77.8	
Odinophagia	Yes	6	-	-	0.335
	No	12	100.0	75.0	
Dysphagia	Yes	12	100.0	68.6	0.192
	No	6	-	-	
Epigastralgia	Yes	6	100.0	50.0	0.038
	No	12	-	-	
Pyrosis	Yes	4	-	-	0.383
	No	14	100.0	76.2	
Body Mass Index (BMI)	≤ 18.5	5	-		0.445
	>18.5	12	100.0	77.8	
Comorbidity	Absent	11	-	-	0.116
	Present	7	100.0	62.5	
Second primary tumor	Yes	5	-	-	0.383
	No	13	100.0	76.2	
Tumor size	≤ 2.0 cm	6	100.0	80.0	0.886
	> 2.0 cm	10	100.0	80.0	
Histological type	Squamous cell carcinoma	16	100.0	90.0	0.247
	Adenocarcinoma	2	100.0	50.0	
Stage (TNM)	0	3	-	-	0.383
	1	15	100.0	76.2	
Surgery	Yes	13	100.0	87.5	0.351
	No	5	100.0	66.7	
Radiotherapy	Yes	6	100.0	66.7	0.351
	No	12	100.0	87.5	
Chemotherapy	Yes	2	100.0	0.0	<0.001
	No	16	-	-	
Total		18	100.0	82.5	

From 18 patients, 6 (33.3%) died during the 36 months follow up, five of them between one to 31 months after diagnosis (1, 4, 5, 14 and 31 months, respectively), and one patient died for other causes than esophageal cancer. Second malignant tumor occurred in 5 (27.8%) cases, one lung cancer (20.0%), one prostate cancer 20.0%, two head and neck cancer (40.0%) and one non-melanoma skin cancer (20.0%), but these tumors did not influence the survival rates in 36 months follow up. None of the patients who died before 36 months had second primary tumor.

Global survival rates probabilities were 76.7% and 64.4% after 12 and 36 months follow up, respectively (Table 3).

**Table 3:** Global Survival Rates Probabilities in Patients With Insipient Esophageal Cancer (Clinical Stage 0 and I - TNM) From Barretos Cancer Hospital, Diagnosed From 1996 To 2005

Variable	Category	n	Global survival rates probabilities (%)		p (Log-Rank)
			12 months	36 months	
Gender	Male	15	73.3	59.3	0.302
	Female	3	-	-	
Age	≤ 49 years old	4	100.0	66.7	0.747
	≥ 50 years old	14	71.4	64.3	
Schooling	Never	5	53.3	53.3	0.628
	Basic incomplete	11	81.8	72.7	
	Basic complete/ High school	2	100.0	50.0	
Tabagism	Never and/or former smoker	6	100.0	80.0	0.188
	Current smoker	9	50.8	50.8	
Etilism	Never	6	83.3	66.7	0.584
	Ever	10	67.5	54.0	
Weight loss	Yes	7	57.1	42.9	0.081
	No	11	90.0	80.0	
Odinophagia	Yes	6	83.3	66.7	0.869
	No	12	73.3	64.2	
Dysphagia	Yes	12	73.3	53.5	0.294
	No	6	83.3	83.3	
Epigastralgya	Yes	6	80.0	60.0	0.956
	No	12	75.0	66.7	
Pyrosis	Yes	4	-	-	0.191
	No	14	71.4	56.3	
Body Mass Index (BMI)	≤ 18.5	5	60.0	60.0	0.443
	>18.5	12	90.9	72.7	
Comorbidity	Absent	11	70.7	60.6	0.599
	Present	7	85.7	68.6	
Second primary tumor	Yes	5	60.0	60.0	0.596
	No	13	83.3	65.6	
Tumor size	≤ 2.0 cm	6	83.3	83.3	0.315
	> 2.0 cm	10	78.8	54.0	
Histological type	Squamous cell carcinoma	16	73.7	67.0	0.858
	Adenocarcinoma	2	100.0	50.0	
Stage (TNM)	0	3	-	-	0.202
	1	15	71.8	56.5	
Surgery	Yes	13	75.0	65.6	0.757
	No	5	80.0	60.0	
Radiotherapy	Yes	6	66.7	50.0	0.306
	No	12	81.8	71.6	
Chemotherapy	Yes	2	73.7	67.0	0.858
	No	16	100.0	50.0	
Total		18	76.7	64.4	

## Discussion

The results herein reported have shown similar socio-demographic characteristic to the Japanese [14, 15] and Korean data [16]. The small number of patients included in the casuistic reflects, in part, the recurrent difficulties to reach patients with incipient esophageal cancer. We investigated 16 years of casuistic and from 1,853 patients only 18 (1.0%) have had early esophageal cancers.

This is very alarming because the vast majority of the patients have advanced esophageal cancer stage at the time of diagnosis, due to the lack of symptoms in the early stages [5]. For the 18 patients of this study, the symptoms (dysphagia, epigastralgia and loss of weight) differed from Dutch patients who predominantly presented gastroesophageal reflux disease (69%), and dysphagia (9%) and epigastralgia (19%) in very small proportion in comparison to Brazilian patients [17]. Also, Dutch patients have showed 36 months of free disease interval in 88% of the patients, similar to data observed in our study [17].

Comparatively, the free disease interval in five years for Japanese patients was 93%, slightly higher to the 82.5% observed Brazilian casuistic in three years [15]. Additionally, Japanese patients showed 7.2% of recurrence in five years of follow up [8], different from Brazilian casuistic which in five years revealed 22.2% in the same period. Interestingly Pech et al reported that the only parameter statistically independent to predict recurrence is the multifocal lesions, which we failed to demonstrate [12].

The overall results clearly demonstrated that survival rates and free disease interval are not associated to the socio-demographic and clinical characteristics. The five years survival rates for all clinical stages in Singapore showed 3% and 6% for male and female, respectively [10] and relative survival rates for Netherlands were 7% and 18%, Italy 6% and 13%, Slovakia Republic 7% and 16% and Denmark 2% and 9% [9]. These values reinforce the aggressiveness of esophageal cancer and endorse the necessity for early cancer detection and cancer prevention. The figures of global survival rates from patients with incipient tumor ratify this premise. German numbers showed survival rates of 83.4% for precancerous adenocarcinoma and 62.9% for invasive squamous cells carcinoma [18], and 77% after 5 years in USA [19] . Korea data showed a global survival rate of 84.3% [16], and Japan 67% for initial cancer stages (T1 and Tis) [14]. However, Japanese studies have also showed five years survival rates ranging from 73% to 80% [8, 15]; and for China a slight superior index of 86% was reported [20]. These numbers do not have a parallel in our study since we demonstrated worse survival rates along the patients follow up. This is a very disturbing observation because reflect, in part, a possible lack of preventive treatment or difficulties to access specialized medical care. Actually, the majority of the cases analyzed in this study showed an advanced stage of cancer, which certainly reduces the advantage of an efficient treatment. Even patients with initial stage of esophageal cancer have showed some homeostatic disorders since weight decrease of the patients was the most prevalent symptom reported.

Good prognosis parameters, such as age under 65 years old, female and tumor with size less than 5 cm did not correlate with our group of patients. On the other hand, well-established parameters of worse prognosis factors include loss of weight in Karnofsky scale [21, 22], which strongly corroborated our findings. Additionally, we observed that patients with squamous cell carcinoma have had a three years global survival rate superior than those with adenocarcinoma (67.0% versus 50.0%, respectively), inversely different from the findings reported by Stein et al [13].

Also, the present study did not corroborate previous reports where the type of treatment influenced the global survive rates. None of the treatment options used for our patients demonstrate any superior performance for this particular proposal. Surgical complications are believed to be one of the most important limitations for good treatment response [19]. Clearly, surgical treatment for initial stages demonstrated better survival rates [7]. Nonetheless, surgeries for initial stages are less invasive which diminish the post-surgical complication [18]. This is promising result corroborated by the results obtained by the endoscopic surgeries for superficial esophageal carcinomas [7, 8, 12, 23, 24].

In conclusion, the main characteristics of Brazilian patients with initial esophageal cancer were comparable to the other countries, including the rates of free disease interval. However, the global survival rates were importantly inferior to those observed elsewhere. Our results, however, should be taking in account very cautiously because of the small number of incipient esophageal carcinomas studied. Moreover, the discrepancies herein reported suggest that the problem is strongly associated to the advanced stage of disease at the moment of diagnosis, which significantly reduce the opportunities for better figures in terms of global survival rates.
